# Cognitive deficits and cortical volume loss in COVID‐19‐related hyposmia

**DOI:** 10.1111/ene.16378

**Published:** 2024-06-08

**Authors:** Haşim Gezegen, Ulaş Ay, Bedia Samancı, Elif Kurt, Sanem Sultan Yörük, Alpay Medetalibeyoğlu, Cömert Şen, Erdi Şahin, Mehmet Barbüroğlu, Faruk Uğur Doğan, Başar Bilgiç, Haşmet Hanağası, Hakan Gürvit

**Affiliations:** ^1^ Behavioral Neurology and Movement Disorders Unit, Department of Neurology, Istanbul Faculty of Medicine Istanbul University Istanbul Turkey; ^2^ Neuroimaging Unit, Istanbul University Hulusi Behçet Life Sciences Research Laboratory Istanbul Turkey; ^3^ Department of Neuroscience Istanbul University Aziz Sancar Institute of Experimental Medicine Istanbul Turkey; ^4^ Department of Internal Medicine, Istanbul Faculty of Medicine Istanbul University Istanbul Turkey; ^5^ Department of Otolaryngology, Head and Neck Surgery, Istanbul Faculty of Medicine Istanbul University Istanbul Turkey; ^6^ Department of Radiology, Istanbul Faculty of Medicine Istanbul University Istanbul Turkey

**Keywords:** cortical atrophy, COVID‐19, hyposmia, MRI, neurological impact

## Abstract

**Background and purpose:**

Studies have found that up to 73% of COVID‐19 patients experience hyposmia. It is unclear if the loss of smell in COVID‐19 is due to damage to the peripheral or central mechanisms. This study aimed to explore the impacts of COVID‐19‐induced hyposmia on brain structure and cognitive functions.

**Methods:**

The study included 36 hyposmic (h‐COV) and 21 normosmic (n‐COV) participants who had recovered from mild COVID‐19 infection, as well as 25 healthy controls (HCs). All participants underwent neurological examination, neuropsychiatric assessment and Sniffin’ Sticks tests. High‐resolution anatomical images were collected; olfactory bulb (OB) volume and cortical thickness were measured.

**Results:**

Addenbrooke's Cognitive Examination—Revised total and language sub‐scores were slightly but significantly lower in the h‐COV group compared to the HC group (*p* = 0.04 and *p* = 0.037). The h‐COV group exhibited poorer performance in the Sniffin’ Sticks test terms of discrimination score, identification score and the composite score compared to the n‐COV and HC groups (*p* < 0.001, *p* = 0.001 and *p* = 0.002 respectively). A decrease in left and right OB volumes was observed in the h‐COV group compared to the n‐COV and HC groups (*p* = 0.003 and *p* = 0.006 respectively). The cortical thickness analysis revealed atrophy in the left lateral orbitofrontal cortex in the h‐COV group compared to HCs. A significant low positive correlation of varying degrees was detected between discrimination and identification scores and both OB and left orbital sulci.

**Conclusion:**

Temporary or permanent hyposmia after COVID‐19 infection leads to atrophy in the OB and olfactory‐related cortical structures and subtle cognitive problems in the long term.

## INTRODUCTION

The SARS‐CoV‐2 coronavirus is a highly contagious RNA virus that may cause a wide range of respiratory symptoms, ranging from mild upper respiratory tract symptoms to severe dyspnea and death [1]. Although COVID‐19 is primarily a respiratory condition, as the pandemic progressed, the spectrum of the symptoms widened as well. From the start of the epidemic, reports of an abrupt loss of smell and taste in SARS‐CoV‐2‐infected patients were documented across the world. The percentage of patients showing anosmia or ageusia was especially high amongst otherwise asymptomatic patients and those experiencing mild levels of other symptoms [2, 3].

A systematic review reported the prevalence of olfactory dysfunction a minimum of 19% and a maximum of 73.6% [4]. In a study by Kaye et al., anosmia was reported to occur in about 73% of patients, mostly women and younger individuals. Additionally, anosmia was the first symptom in 27% of these patients [5]. In a case–control study, where the infection was still active for the majority of the patients (72%), smell impairment was identified in 67% of the patients [6]. However, a case–control study with 2‐year follow‐up showed that 42% and 28% of the patients were hyposmic 1 and 2 years after the infection, respectively [7].

The question of whether the loss of smell associated with COVID‐19 is central or peripheral has been questioned since the first appearance of olfactory symptoms. The axons of olfactory sensory neurons coalesce to form the olfactory nerves and project to the ipsilateral olfactory bulb (OB). The OB is a six‐layered structure within the central nervous system (CNS) whose targets are the primary olfactory cortices (POC) via the lateral olfactory tract (LOT). Whilst the olfactory system sticks out as the only sensory modality without a thalamic relay, the OB can be considered analogous to a thalamic relay, since its direct target is the POC [8]. POCs, which are the direct targets of the OB via the LOT, are the following: anterior olfactory nucleus, olfactory tubercle, piriform cortex, the superficial cortex‐like region of the amygdala, which is the olfactory amygdala (consisting of the three nuclei that are the cortico‐amygdaloid transitional area, anterior amygdaloid area and the cortical nucleus), and finally the rostral entorhinal cortex [9, 10]. Four of the POCs with the exception of the OT are directly and the latter is indirectly via ventral striatum connected to secondary olfactory cortices [11].

In a review on the cerebral invasion of COVID‐19, axonal transport via the olfactory nerve, initially reaching the olfactory cortices and then spreading to neighboring structures, was mentioned as the principal direct route [12]. In a longitudinal study of a large sample from the UK, it was shown that people who had been infected with SARS‐CoV‐2 had significantly less gray matter thickness in the left parahippocampal gyrus and the lateral orbitofrontal cortex, functional connectivity decreases in limbic brain regions forming a mainly olfactory network and longitudinally greater cognitive decline compared to healthy controls [13]. In the same study a separate analysis revealed that the individuals who had been hospitalized with COVID‐19 exhibited a more widespread pattern of greater reduction in gray matter thickness in the fronto‐parietal and temporal regions compared to non‐hospitalized patients.

Different hypotheses have been put forward to explain the smell dysfunction in COVID‐19 patients: (i) rhinorrhea, nasal obstruction and congestion; (ii) loss of olfactory receptor neurons; (iii) damage to the olfactory epithelium's support cells; and (iv) brain invasion that impacts the olfactory centers [14, 15]. Rather than being mutually exclusive hypotheses it is highly likely that all four are simultaneously valid.

The main purpose of this prospective, case–control study is to investigate the effects of hyposmia/anosmia caused by COVID‐19 infection on structural magnetic resonance imaging (MRI) changes and cognitive impairment. Our hypothesis is that participants with hyposmia would have atrophy in the OB and olfactory‐related cortical structures, leading to cognitive domain impairments.

## METHODS

### Participant selection

The study protocol was approved by the local ethics committee (approval no. 102157), and written informed consent was obtained from all participants. Participants were invited via free social media platforms of the authors and their close circle (WhatsApp, Twitter or Instagram) and the Istanbul Faculty of Medicine COVID‐19 follow‐up outpatient clinic between May 2021 and December 2022.

The patients who were diagnosed with COVID‐19 with mild symptoms and verified by positive results on a polymerase chain reaction (PCR) test on a nasopharyngeal swab sample were included as the patient group. None of the patients was hospitalized, had severe respiratory difficulties or needed assistance at home. Patients who had hyposmia/anosmia with or without other COVID‐19 symptoms were classified as the hyposmic COVID‐19 group (h‐COV) whilst patients who did not exhibit any symptoms of olfactory impairment during or after the COVID‐19 infection were classified as the normosmic COVID‐19 group (n‐COV). Participants without a history of COVID‐19 symptoms or positive PCR test results were included as the healthy control (HC) group. All groups were matched by age, sex, education and smoking status.

Inclusion criteria for all groups were (i) 16–65 years old and (ii) able to perform olfactory and cognitive assessment. Exclusion criteria were (i) not able to perform olfactory and cognitive assessment, (ii) contraindication for MRI scan, (iii) history of or present neurological disease causing alterations in olfaction or cognition (e.g., any neurodegenerative disease, cerebrovascular disease, multiple sclerosis, head trauma etc.), (iv) abnormal neurological findings on examination, (v) history of nose surgery or severe nose trauma and (vi) history of or current diagnosis of depression, anxiety and other mood disorders. All participants were recruited following an otolaryngology examination to include only participants who did not have any olfactory dysfunction due to allergic, traumatic, surgical, tumor‐related causes or side effects of medications.

### Clinical, neuropsychological and neuropsychiatric assessment

Neurological examination was performed by an experienced neurologist. Patients were asked for any neurological complaints since COVID‐19. Findings on physical examination were classified into meningism, cranial nerve abnormalities, pyramidal and extrapyramidal signs, cerebellar abnormalities, sensory signs and gait abnormalities.

Cognitive evaluation was performed by a neuropsychologist. Addenbrooke's Cognitive Examination—Revised (ACE‐R), which also includes Mini‐Mental State Examination (MMSE) items for general cognitive screening [16], Free and Cued Selective Reminding Test (FCSRT) for evaluating verbal memory [17], Beck Depression Inventory (BDI) [18] for evaluating depression and Beck Anxiety Inventory (BAI) [19] for evaluating anxiety, were performed. BDI values of 17 and above were considered clinically significant for depression. A total score of 8–15 was considered mild, 15–25 was moderate and 26–63 was severe for BAI.

### Olfactory assessment

A licensed otolaryngologist conducted a thorough standardized clinical and rhinological examination on each participant to assess for any pathologies affecting their sense of smell like sinonasal disease, trauma and upper respiratory tract infection.

A Turkish version of the sinonasal outcome test 22 (SNOT‐22) was used to assess how COVID‐19 affected sinonasal symptoms [20]. Olfactory tests were performed using the Sniffin’ Sticks test battery (Burghart Messtechnik, Germany), which has three components to assess olfactory threshold (T), discrimination (D) and identification (I). Each component has a scale of 16, and TDI is a composite score representing the sum of these three scores. Normosmia is defined for scores ≥30.5, hyposmia for scores between 16.5 and 30.5, and anosmia for scores <16.5 [21].

### MRI acquisition

Neuroimaging data were collected with a 32‐channel head coil on a Phillips‐Achieva 3.0 T scanner installed at Istanbul University Hulusi Behçet Life Sciences Research Laboratory. Two different T1‐weighted images were obtained with turbo field echo sequence and T2‐weighted images were obtained with turbo spin echo sequence. The acquisition parameters of T1‐weighted images were as follows: repetition time (TR) 8.2 ms, echo time (TE) 3.2 ms, field of view (FOV) 256 × 256 mm, flip angle 7°, 176 sagittal slices, slice thickness 1 mm, voxel size 1 × 1 × 1 mm; and TR = 8.4 ms, TE = 3.9 ms, FOV = 250 × 250 mm, flip angle 8°, 180 sagittal slices, slice thickness 1 mm, voxel size 1 × 1 × 1 mm. The acquisition parameters of T2‐weighted images were as follows: TR = 3000 ms, TE = 80 ms, FOV = 200 × 205 mm, flip angle 90°, 55 coronal slices, slice thickness 2 mm, voxel size 0.57 × 0.72 × 2 mm.

### Olfactory bulb volume quantification

Olfactory bulb volume was calculated by manual segmentation of the OBs using ITK‐SNAP Software v. 3.8 (University of Pennsylvania and University of Utah, www.itksnap.org) [22]. Olfactory bulb morphology was evaluated on high‐resolution coronal T2 sections. After a training period, the left and right OBs were segmented by two blinded and independent raters (BS, UA) for 20 participants. The inter‐rater segmentation reliability scores with intraclass correlation coefficient for volumes for two raters were 0.86 for the left OB and 0.931 for the right OB. After that, all OBs were segmented by these two raters.

### Anatomical image processing

FreeSurfer (version 7.3.2) software (http://surfer.nmr.mgh.harvard.edu/) was used to detect vertex‐based cortical thickness (CT) differences on the whole brain of the studied groups. As described above, two different T1‐weighted MRI images from each participant were analyzed using the standard recon‐all pipeline in FreeSurfer, as previously reported [23]. Using multiple T1 images in FreeSurfer offers several advantages. First, employing multiple T1 images enhances the accuracy and robustness of structural brain segmentation and cortical surface reconstruction processes [24, 25]. Secondly, utilizing more than one T1 image allows for better correction of motion artifacts and intensity inhomogeneities, thereby improving the quality of the final segmentation results [25, 26]. Moreover, multiple T1 images enable the detection and correction of potential scanner‐related biases or inconsistencies, leading to more reliable and reproducible outcomes [25]. Overall, two different T1 images were used because the utilization of multiple T1 images in FreeSurfer contributes to increased accuracy, robustness and reliability in structural brain analysis. FreeSurfer processing took roughly 5–6 h on our PC workstation running Ubuntu 22.04.2 with an Intel® Core(™) i7‐6700K processor and 32 GB of DDR4 memory.

After the recon‐all stage is completed, outputs of the cortical analysis were quality checked by visual inspection and it was concluded that no troubleshooting was necessary. The design matrices were created by a FreeSurfer Group Descriptor File (https://surfer.nmr.mgh.harvard.edu/fswiki/FsgdExamples). The CT maps of all patients were projected onto the FsAverage template, which is based on the MNI305 template [27]. The thickness maps were smoothed at a 15 mm Gaussian kernel [28, 29].

## STATISTICAL ANALYSIS

The SPSS package (v.26) was used to evaluate demographic and clinical data. First, the conformity of the data to the normal distribution was evaluated with the Shapiro–Wilk test. One‐way analysis of variance (ANOVA) and afterwards the Bonferroni multiple comparison test were used to compare normally distributed variables, whilst the Kruskal–Wallis *H* test and afterwards the Tamhane's T2 multiple comparison tests were used to compare non‐normally distributed variables. The Pearson chi‐squared test was used to compare categorical variables. Continuous variables that conformed to the normal distribution were presented as mean (standard deviation), and the non‐normally distributed continuous variables were expressed as median (interquartile range, IQR). The significance level was accepted as *p* < 0.05.

Two separate multivariate analyses of covariance (MANCOVA) were performed to compare Sniffin’ Sticks scores and OB volumes. In the comparison of Sniffin’ Sticks scores, the scores of the test were included in the analysis as the dependent variable, group as fixed factor and age, gender and education as covariates. In the comparison of OBs between groups, OB volumes were determined as the dependent variable, group as fixed factor, and age, sex, education and estimated total intracranial volume obtained from FreeSurfer in order to control for the head size of the participants were determined as covariates. The Bonferroni multiple correction test was used to compare the estimated marginal means of the groups in both MANCOVAs and the significance level was accepted as *p* < 0.05.

Differences in CT measurements were compared across groups using the general linear model implemented in FreeSurfer (mri_glmfit). The cluster‐wise corrections for multiple comparisons were performed by running a Monte Carlo simulation with 10,000 iterations, a cluster‐forming threshold of *p* < 0.001, a cluster‐wise threshold set at *p* < 0.05 [30, 31] and Bonferroni corrected for the two hemispheres. Statistically significant clusters of group comparisons were superimposed on the FsAverage surface.

## RESULTS

### Clinical and demographic characteristics

The hyposmic COVID‐19 group had 36 participants, with an average age of 34.42 ± 11.01 years, and the n‐COV group had 21 participants, with an average age of 32.90 ± 9.03 years. Lastly, the HC group had 25 participants, with an average age of 31.80 ± 7.93 years. Other demographic characteristics were similar in all groups. All of the participants had an active working life and at least a university degree. Socio‐demographic, clinical features, cognitive and physiological test results of groups are reported in Table 1.

**TABLE 1:** Demographic and neuropsychological results.

	h‐COV (*n* = 36)	n‐COV (*n* = 21)	HCs (*n* = 25)	Statistic	*p*	Post hoc
Age (years), mean ± SD	34.4 (11.01)	32.9 (9.03)	31.8 (7.93)	0.557^a^	0.575	–
Sex (M/F), *n*	15/21	5/16	14/11	4.874^b^	0.087	–
Education (years), mean (SD)	16.06 (2.16)	17.14 (1.49)	16.72 (1.51)	2.477^a^	0.091	–
Smoking (Y/N), *n*	21/15	7/14	7/18	1.257^b^	0.534	–
SNOT‐22 score, median (IQR)	9.00 (21.50)	12.00 (20.00)	7.00 (8.00)	1.783^c^	0.410	–
Time after COVID (days), median (IQR)	495.50 (321.75)	105.00 (231.50)	NA	2.694^d^	**0.007**	HC < h‐COV
BDI, median (IQR)	4.00 (8.50)	4.00 (5.00)	5.00 (4.00)	0.299^c^	0.861	–
BAI, median (IQR)	0.00 (0.00)	0.00 (0.00)	0.00 (0.00)	0.764^c^	0.683	–
MMSE, median (IQR)	30.00 (1.00)	29.00 (2.75)	29.00 (2.00)	4.974^c^	0.083	
FCSRT, Cueing index, mean (SD)	0.95 (0.08)	0.97 (0.06)	0.97 (0.06)	0.703^a^	0.498	–
ACE‐R total, mean (SD)	92.20 (5.07)	92.79 (6.02)	95.45 (3.26)	3.238^a^	**0.045**	h‐COV < HC
ACE‐R attention and orientation, median (IQR)	17.50 (2.00)	18.00 (2.00)	18.00 (1.00)	2.807^c^	0.246	–
ACE‐R memory, median (IQR)	23.00 (2.25)	23.00 (5.00)	23.00 (4.00)	2.839^c^	0.242	–
ACE‐R fluency, median (IQR)	13.00 (2.00)	13.00 (2.00)	14.00 (1.00)	4.392^c^	0.111	–
ACE‐R language, median (IQR)	24.00 (1.25)	25.00 (2.00)	26.00 (1.00)	6.592^c^	**0.037**	h‐COV < HC
ACE‐R visuospatial, median (IQR)	15.00 (1.00)	15.00 (1.00)	16.00 (1.00)	2.179^c^	0.336	

*Note*: Data are presented as mean (standard deviation) or median (interquartile range). All bold *p* values are statistically significant ones.

Abbreviations: ACE‐R, Addenbrooke's Cognitive Examination—Revised; BAI, Beck Anxiety Inventory; BDI, Beck Depression Inventory; F, female; FCSRT, Free and Cued Selective Reminding Test; h‐COV, hyposmic COVID participants; HCs, healthy controls; M, male; MMSE, Mini‐Mental State Examination; N, no; n‐COV, normosmic COVID participants; SNOT‐22, sinonasal outcome test 22; Y, yes; IQR, interquartile range; NA, not applicable.

^a^ One‐way ANOVA.

^b^ Pearson chi‐squared test.

^c^ Kruskal–Wallis *H* test.

^d^ Mann–Whitney *U* test.

The participants in the h‐COV group were enrolled in the study 495.50 (median) days after COVID‐19 infection, whilst the participants in the n‐COV group were enrolled 105.00 days after COVID‐19 infection (*p* = 0.001).

Amongst the participants in the h‐COV group, 14 individuals had ongoing hyposmia complaints at the time of enrollment. The hyposmia complaints of the other 22 patients had lasted for 61.2 ± 74.5 days. A total of five individuals had concurrent cacosmia (foul odor) along with hyposmia.

In both the h‐COV and control groups, two individuals had a history of allergic rhinitis. Before conducting the olfactory test, an ear, nose and throat examination was performed. Amongst these two groups, one patient with active allergic rhinitis symptoms was detected from each group and received a two‐week topical steroid treatment prior to the olfactory tests.

### Neuropsychological and neuropsychiatric scale results

Mini‐Mental State Examination scores did not differ between groups (*p* = 0.081). ACE‐R scores, although within normal range, were slightly but significantly lower in the h‐COV group (mean 92.2 ± 5.07) compared to the HC group (mean 95.45 ± 3.26; corrected *p* = 0.049). An ACE‐R subgroup analysis revealed a significant difference only in the language sub‐score between the h‐COV (median 24 ± 1.25) and HC (median 26 ± 1.0; corrected *p* = 0.045) groups. The n‐COV group had no statistically significant difference with the h‐COV group and HC group in terms of ACE‐R scores (*p* = 0.91 and *p* = 0.18, respectively). The Cueing index of the FCSRT, which is a sensitive measure of limbic‐type memory impairment, did not show any significant difference between the groups (*p* = 0.498).

Based on the BDI scores, three participants in the h‐COV group (18, 18 and 21 points), two participants in the n‐COV group (20 and 23 points) and one participant in the HC group (17 points) scored above the cut‐off for depression. According to the BAI scores, one participant from both the h‐COV (11 points) and n‐COV groups (9 points) scored above the cut‐off for mild anxiety, whilst no one in the HC group scored above the cut‐off. There were no significant differences in the total scores of BDI and BAI between the three groups (*p* values were 0.718 and 0.575, respectively).

### Olfactory scale and test results

The median and IQR of the SNOT‐22 scores were found to be 9.00 (IQR 21.50) in the h‐COV group, 12.00 (IQR 20.00) in the n‐COV group and 7.00 (IQR 8.00) in the HC group. There was no significant difference observed amongst the groups (*p* = 0.41).

The results of the Sniffin’ Sticks test are shown in Table 2. The h‐COV group exhibited poorer performance in terms of D, I and TDI scores compared to the n‐COV and HC groups after adjusting age, sex and education (*p* < 0.001, *p* = 0.001 and *p* = 0.002 respectively). For the T score, no significant difference was found between the three groups (*p* = 0.828).

**TABLE 2:** Sniffin’ Sticks test results.

	h‐COV (*n* = 36)	n‐COV (*n* = 21)	HCs (*n* = 25)	*F*	*p*	*η*_p_^2^	Post hoc
Threshold score (T)	7.90 (0.44)	8.27 (0.57)	7.80 (0.52)	0.195	0.823	0.005	–
Discrimination score (D)	12.04 (0.24)	13.76 (0.31)	14.06 (0.28)	17.588	**< 0.001**	0.331	h‐COV < HC h‐COV < n‐COV
Identification score (I)	11.99 (0.26)	13.49 (0.34)	13.31 (0.31)	8.118	**0.001**	0.186	h‐COV < HC h‐COV < n‐COV
TDI score	31.98 (0.67)	35.47 (0.88)	35.28 (0.79)	7.065	**0.002**	0.166	h‐COV < HC h‐COV < n‐COV

*Note*: Data are presented as mean (standard error). All scores adjusted for age, sex and education. All bold *p* values are statistically significant ones.

Abbreviations: h‐COV, hyposmic COVID‐19 group; HCs, healthy controls; n‐COV, normosmic COV‐19 group.

### Structural MRI results

#### Olfactory bulb volume results

**FIGURE 1:**
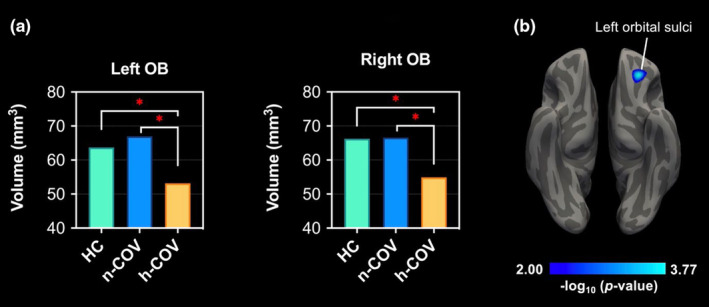
(a) Comparison of OB volumes of the three groups. (b) As a result of whole brain CT analysis, the region showing a decrease in CT in the hyposmic COVID group compared to healthy controls. *p < 0.05.

Left and right OB volumes are presented in Table 3. A noticeable decrease in both left and right OB volumes was observed in the h‐COV group compared to the n‐COV and HC groups (*p* value 0.003 for left OB, 0.006 for right OB) **(**Figure 1a **)**.

**TABLE 3:** Olfactory bulb (OB) volumes.

	h‐COV (*n* = 36)	n‐COV (*n* = 21)	HCs (*n* = 25)	*F*	*p*	*η*_p_^2^	Post hoc
Left OB	53.18 (2.78)	66.97 (3.59)	63.73 (3.24)	5.394	**0.007**	0.129	h‐COV < Control h‐COV < n‐COV
Right OB	54.92 (2.79)	66.59 (3.59)	66.26 (3.24)	4.478	**0.012**	0.115	h‐COV < Control h‐COV < n‐COV

*Note*: Data are presented as mean (standard error). OB volumes corrected for age, sex, education and total intracranial volume. All bold *p* values are statistically significant ones.

Abbreviations: h‐COV, hyposmic COVID‐19 group; HCs, healthy controls; n‐COV, normosmic COV‐19 group.

#### Whole brain cortical thickness analysis results

In whole brain CT analysis, a decrease in CT in the left orbital sulci was observed in the h‐COV group compared to HCs. However, no significant difference was found between the n‐COV and the h‐COV groups **(**Figure 1b **)**.

When the subgroup of 14 individuals with ongoing olfactory loss was compared with the HC group, no additional CT difference was found other than the left orbital sulci. Also, there was no CT difference between participants with ongoing olfactory loss and those with transient olfactory loss in the h‐COV group.

#### Correlations between clinical and imaging results

There was no relationship between OB volume and the volume of the left lateral orbital gyrus. Also no significant correlation was found between SNOT‐22 and the T, D, I and TDI scores. However, there were significant positive correlations between the D score and the volume of the left OB, right OB and left orbital sulci (*r* = 0.259, *p* = 0.020, *r* = 0.288, *p* = 0.010, and *r* = 0.231, *p* = 0.039 respectively). Also, positive correlations were found between the D score and left and right OB volumes (*r* = 0.259, *p* = 0.020, and *r* = 0.288, *p* = 0.010), and between the I score and the volume of the right OB (*r* = 0.317, *p* = 0.004). There were no significant correlations between ACE‐R total or sub‐scores with olfactory cortical regions or OB volumes (*p* > 0.05).

## DISCUSSION

This study is the first case–control study to evaluate the objective olfactory and cognitive tests, OB volume and CT together in individuals experiencing COVID‐19‐related olfactory loss on a long‐term basis. The present findings suggest that temporary or permanent hyposmia after COVID‐19 infection leads to impairment of olfactory discrimination and identification, decreased OB volumes, and reduced CT in the left orbital sulci, specifically in the h‐COV group. No CT differences were found in the other olfactory‐related cerebral structures. Moreover, the decreased volumes of both right and left OB in the h‐COV group were also below the atrophy cut‐off of 58 mm^3^ for younger than 45‐year‐old normal subjects (in contrast, volumes were over the cut‐off in the n‐COV and HC group; see Table 3) [32]. Although none of the participants, including those of the h‐COV group, had real‐life cognitive complaints in general and language problems, such as word‐finding difficulties in particular, and no different MMSE scores, h‐COV group's total ACE‐R (max: 100) and language sub‐scores (max: 26) were significantly lower than those of the HC group (92.2 and 24 vs. 95.44 and 26) and the total score difference was seemingly mainly driven by the language sub‐scores as there were no differences between other sub‐scores. A closer inspection of the individual items of the test suggested that the difference mainly stemmed from the lower performance in the 12‐point naming section of the language sub‐test, although this was not specifically subjected to statistical analysis.

As mentioned above, in the UK longitudinal study where 351 participants were evaluated with MRI an average of 141 days after COVID‐19 infection, longitudinal gray matter loss was demonstrated in limbic cortical regions directly associated with the olfactory and memory processing systems (especially in the orbitofrontal cortex and parahippocampal gyrus) [13]. However, no difference in OB volumes was observed between the COVID‐19 and the HC groups. The lack of difference may be related to the study design, since it is noteworthy that no distinction was made between the patients with hyposmia and normosmia in the COVID‐19 patient group in that study. In another imaging study involving 27 COVID‐19‐related hyposmic individuals and 18 HCs, where no difference was found between the OB volumes, interestingly increased functional and structural connectivities were found in the patient group [33]. The connectivity increases (functional connectivity with the anterior piriform cortex and structural with the medial orbitofrontal cortex) were interpreted as the compensatory response of the olfactory neural network to the relatively recent COVID‐19 infection (10–76 [31.8 ± 21.0] days). In the patients' 1‐year follow‐up study the increase in the structural connectivity was found to be not significant [34]. Our findings of decreased volumes of both OBs and the left secondary‐olfactory‐cortex‐related area might reflect the failure of compensation with persistent olfactory loss during a relatively late period, since the mean days after the infection were 402 ± 215.8 days in our h‐COV group.

The duration of olfactory loss in individuals with acquired olfactory dysfunction (post‐infectious, chronic inflammation, idiopathic and congenital) was found to be associated with a more significant loss of gray matter in the gyrus rectus and orbitofrontal cortex [35]. Additionally, no significant differences were found between permanent and temporary olfactory impairment in h‐COV group. These results may indicate that COVID‐19‐related olfactory loss causes changes in the brain even when the olfactory loss is temporary. However, these results may change in longitudinal studies with longer follow‐up periods, and different affected regions may be found in patients with permanent and temporary olfactory loss.

Many studies [13, 35] have shown that cortical atrophy following the loss of smell predominantly affects the left olfactory cortex. The brain shows specialization for different aspects of smell perception in the right and left hemispheres, similar to many other brain functions [36, 37]. In numerous studies, atrophy in the left olfactory cortex has been observed in healthy elderly individuals and those with Alzheimer's dementia, which can be explained by the principle that the thicker of the two homotopic cortices thins faster [38]. Our finding of cortical thinning in the left orbital sulci in the h‐COV group can be interpreted accordingly.

Subjects with SARS‐CoV‐2 infection exhibited a faster cognitive decline over time [13]. Patients who reported both dysgeusia and hyposmia during the acute phase of COVID‐19 showed less improvement in verbal memory tests over time compared to patients without dysgeusia/hyposmia [39]. Moreover, a correlation between cognitive impairment and reduced fluorodeoxyglucose uptake in the frontoparietal regions was observed in 29 subacute stage COVID‐19 patients, who were cognitively normal before the infection by fluorodeoxyglucose‐positron emission tomography [40]. In a community‐based prospective study of “dementia‐free” elderlies, researchers discovered that olfactory impairment was linked to accelerated cognitive decline and reduced volume in brain regions like the fusiform gyrus, middle temporal cortex, hippocampus and entorhinal cortex; these findings were interpreted as suggesting that the olfactory impairment could serve as a predictor for future cognitive decline and an indicator of neurodegeneration in the brain [41].

One recent comprehensive review evaluating the cognitive effects of COVID‐19 reported that “Memory, attention, and executive functions appeared to be the most affected domains”, language and visuo‐spatial abilities being rarely affected [42]. However, most studies used the MMSE or the Montreal Cognitive Assessment as the cognitive screening instruments, neither of which includes items for a comprehensive language assessment like the ACE‐R does. One study evaluating the cognitive effects of COVID‐19 and using ACE‐R found differences in orientation attention and fluency sub‐score but not in language, visuo‐spatial and memory sub‐scores [43]. However, this study was conducted shortly after recovery from the disease, and the comparison was between individuals already at risk for cognitive decline and those who were not.

There was no correlation between ACE‐R total and ACE‐R language scores with olfactory cortical regions or OB volumes. Yet, since the only demonstrated cortical difference was in the left hemisphere and the only documented cognitive difference was in the language domain, attempting to associate them with each other may not be a too far‐fetched speculation. The orbitofrontal part of the left hemisphere is not generally included in the conception of a linguistic neural network. However, there is growing evidence that it does contribute to linguistic processing. This evidence has been reported in a very recent review stating: “This review demonstrates that not only the linguistic tasks that involve the processing of socially, pragmatically and emotionally relevant information engage orbitofrontal cortex and its neurobiological mechanisms, but also specific receptive and expressive language performances rely on specific neurophysiological properties of this region (e.g., the gray matter volume and the functional activation of orbitofrontal cortex and the uncinate fasciculus that connects orbitofrontal cortex), which in many cases, demand executive functions” [44].

An evaluation of COVID‐19 patients approximately 30 days after hospital discharge revealed high rates of depression, anxiety, insomnia and obsessive‐compulsive behaviors [45]. However, this study was conducted in the early phase, after hospital discharge, on a group of patients with moderate to severe symptoms, which required hospitalization. Considering the psychological burden associated with hospitalization those psychiatric symptoms may be considered as reactive, rather than primary. In a meta‐analysis, the overall impact of the pandemic has been found to be associated with worsening psychiatric symptoms. However, the long‐term effects of direct COVID‐19 infection have been linked to either no or mild symptoms. Studies have shown that the long‐term prevalence of anxiety, depression and sleep disturbances is comparable to the general population level, indicating that the deterioration in mental health could be attributed to the indirect effects of COVID‐19, such as psychosocial factors [46]. No significant difference was found in the scores of anxiety and depression scales between the patients and the HC group in the long term. Importantly, it should be re‐emphasized that there were no hospitalized patients in our cohort, and all patients had mild symptoms.

The T, D, I and TDI scores of the HC group and the n‐COV group in our study were found to be similar to each other, whilst those of the h‐COV group were significantly lower than both groups, with the exception of the T score. Moreover, significant positive correlations were found between TDI score and left olfactory gyrus volume and I score and right OB volume.

The growing evidence suggests that SARS‐CoV‐2 has neurotropic features. Human brain parenchyma and cerebrospinal fluid have both been found to contain the SARS‐CoV‐2 virus; however, it is still unknown how the virus enters these tissues [47]. The possible routes are as follows: neuronal (by moving along cranial nerves such as the vagal, facial, glossopharyngeal, trigeminal and olfactory nerves); systemic (crossing through endothelial cells and gaining entry into cells that cross the blood–brain barrier); and getting entry to areas that contain cerebrospinal fluid [48]. Studies using animal models of OC43 coronavirus infection, which is a coronavirus type and mostly known to cause mild respiratory symptoms, have shown that viral particles were present in the OB as early as 3 days after inoculation, and in the cortex by day 7 [49]. In ACE2 transgenic mice infected with SARS‐CoV‐1, researchers observed a similar pattern of viral entry through the OB, followed by rapid invasion of the CNS [50]. Also in an animal model that investigated post COVID‐19 effects elevated levels of chemokines were detected in both cerebrospinal fluid and serum of mice exhibiting mild respiratory symptoms. These neuroinflammatory changes seemed to trigger the activation of microglia in regions of the hippocampus and subcortical white matter driven by increased levels of chemokine 11 [51]. Also, gray matter loss was demonstrated in limbic cortical regions associated with the olfactory network after COVID‐19 infection suggesting potential mechanisms for the spread of the disease (or the virus itself) in the brain [13]. In our study, atrophy of the OBs and orbital sulci, the impaired neuropsychological test results, and reduced discrimination and identification scores in the Sniffin’ Sticks test in the h‐COV group supported the neurotropic feature of this virus via the olfactory nerve.

It appears that the OB is affected in the early stages of the disease. Experimental and imaging studies in the literature support that cortical structures are not affected before OB involvement [47, 48, 49]. Functional and structural studies have also identified changes in connectivity within olfactory cortices [13, 33]. Our study, in line with the literature, demonstrated that COVID‐19‐related olfactory loss is associated with atrophy in the OB and olfactory‐related cortical structures even in the long term. However, the practical or prospective implications of these findings are yet to be fully understood.

The present study has some limitations. The sample size is relatively small. The participants in the h‐COV (402 ± 215.8 days) and n‐COV (220.2 ± 199.1 days) groups were included in the study at different times after the infection. The reason for this is that, at the beginning of the pandemic, the Alpha and Delta variants were causing more hyposmia, whilst towards the end of the pandemic the emergence of the Omicron variant resulted in less olfactory loss [52]. This global shift in variants led to our participants with olfactory loss being from an earlier period. This situation also brings us to another limitation of our study. It is not known which COVID‐19 variant the patients were infected with. The Omicron variant causes a lower prevalence of olfactory dysfunction [53] and this was confirmed by subsequent large‐cohort studies. The combined average prevalence is 13%, representing a threefold to fourfold decrease from the anosmia prevalence caused by the Alpha and Delta variants (at 35%–50%) [54]. Thus, the differences between the different variants was not evaluated. Additionally, the vaccination status of the participants as well as information about the participants' cognitive status before COVID‐19 were not recorded. The effect of the vaccination on the results could not be evaluated.

## CONCLUSION

This study, in contrast to the majority of case series or cohort studies published so far, does not focus on assessing whole brain volumes and gross abnormalities that could be observed. The main finding of the study is that COVID‐19‐related olfactory loss is associated with atrophy in the OB and olfactory‐related cortical structures in the long term. In individuals with olfactory loss, it is evident that the D and I scores decreased. Moreover, when these findings were considered together with the decline in ACE‐R scores, it becomes apparent that hyposmia induces certain changes in the CNS, regardless of the duration of the olfactory loss. However, whether these changes have practical or prospective implications remains to be fully understood and requires further investigation. Future research should focus on assessing the functional and cognitive consequences of these structural changes and how they may impact the quality of life and overall health outcomes of individuals who experienced COVID‐19‐related olfactory loss. Additionally, prospective studies could help determine the progression and reversibility of these structural changes over time and their potential role in predicting cognitive decline or other neurological conditions. Even though the COVID‐19 pandemic may have ended, the answer to whether it continues to be a public health issue due to its long‐term effects will be provided by these studies.

## AUTHOR CONTRIBUTIONS

**Haşim Gezegen:** Conceptualization; investigation; methodology; writing – review and editing; writing – original draft; project administration. **Ulaş Ay:** Conceptualization; investigation; visualization; writing – review and editing; software; formal analysis; validation; project administration. **Bedia Samancı:** Conceptualization; supervision; writing – review and editing; methodology; funding acquisition; investigation. **Elif Kurt:** Conceptualization; investigation; software. **Sanem Sultan Yörük:** Conceptualization; project administration. **Alpay Medetalibeyoğlu:** Investigation. **Cömert Şen:** Investigation; project administration. **Erdi Şahin:** Conceptualization; project administration; investigation. **Mehmet Barbüroğlu:** Conceptualization. **Faruk Uğur Doğan:** Project administration. **Başar Bilgiç:** Conceptualization; methodology; supervision. **Haşmet Hanağası:** Conceptualization; supervision. **Hakan Gürvit:** Conceptualization; supervision; methodology.

## FUNDING INFORMATION

This study was funded by the Scientific Research Projects Coordination Unit of Istanbul University, project number TSA‐2020‐37171.

## CONFLICT OF INTEREST STATEMENT

The authors declared no potential conflicts of interest with respect to the research, authorship and/or publication of this article.

## Data Availability

Anonymized statistical data to reproduce the main findings are available from the corresponding author upon reasonable request from any qualified investigator. Other data are not available due to their containing information that could compromise the privacy of research participants.
